# Hyoscine butylbromide for colorectal polyp detection: prospective, randomized, placebo-controlled trial

**DOI:** 10.6061/clinics/2017(07)01

**Published:** 2017-07

**Authors:** Carlos Eduardo Oliveira dos Santos, Hamilton Moreira, Julio Carlos Pereira-Lima, Carmen Australia Paredes Marcondes Ribas, Fernanda de Quadros Onófrio, Alexandre Eduardo Augusti Czecko, Rafael Koerich Ramos, Caroline Aragão de Carvalho

**Affiliations:** I Departamento de Endoscopia e Gastroenterologia, Santa Casa de Caridade, Bage, RS, BR; II Programa de Pos graduacao em Principios de Cirurgia, Faculdade Evangelica do Parana, Curitiba, PR, BR; III Departamento de Gastroenterologia e Endoscopia, Hospital Santa Casa, Porto Alegre, RS, BR

**Keywords:** Colonoscopy, Adenoma, Colonic Polyps, Scopolamine Hydrobromide

## Abstract

**OBJECTIVES:**

The removal of pre-malignant colorectal lesions prevents cancer. Hyoscine has been proposed as a means of improving diagnosis by reducing colonic movements. The aim of this study was to analyze whether this anti-spasmodic enhances the detection of pre-malignant colorectal lesions.

**METHODS:**

In a randomized, double-blinded fashion patients received hyoscine or a saline solution in all consecutive colonoscopies in which the cecum was reached. Lesions were analysed with respect to number, size, location, histology and capillary pattern.

**RESULTS:**

A total of 440 colonoscopies were randomized. The overall polyp detection rate (PDR) and the adenoma detection rate (ADR) were 65.2% and 49.3%, respectively. In the hyoscine group, non-polypoid lesions were detected significantly more often (*p*=0.01). In the placebo group 281 lesions were diagnosed (202 adenomas) and in the hyoscine group 282 lesions were detected (189 adenomas) (*p*=0.23). The PDR and ADR were similar between the placebo and hyoscine groups (64% *vs* 66% and 50% *vs* 47%, respectively). No differences were observed between the two groups in the advanced-ADR or advanced neoplasia detection rate, as well the mean numbers of polyps, adenomas, advanced adenomas and advanced neoplasias detected per patient. The administration of hyoscine also did not improve the diagnostic accuracy of digital chromoendoscopy. The presence of adenomatous polyps in the right colon was detected significantly more frequently in the hyoscine group (OR 5.41 95% CI 2.7 - 11; *p*<0.01 *vs* OR 2.3 95% CI 1.1 - 4.6; *p*=0.02).

**CONCLUSION:**

The use of hyoscine before beginning the withdrawal of the colonoscope does not seem to enhance the PDR and the ADR.

## INTRODUCTION

Colorectal cancer (CRC) represents one of the leading causes of cancer-related deaths worldwide. The diagnosis of pre-malignant lesions, and the subsequent removal of such lesions is a well-recognized strategy in the secondary prevention of CRC 1. Colonoscopy is considered the gold standard in achieving this goal, with the adenoma detection rate (ADR) (defined as the index of procedures in which at least one adenoma is diagnosed) considered a known indicator of the quality of the method 2. Nevertheless, a considerable number of adenomas are missed 3,4, which are related to interval cancer, especially in the right colon 5. Interval cancer in the right colon could be partially explained by the non-diagnosis of non-polypoid lesions, since these neoplasms are more common in the right colon and are more aggressive 6.

Many factors could contribute to the failure to diagnose these lesions, including inadequate bowel preparation 7,8, colonoscopy technique 9, colonoscope withdrawal time 10 and polyp location 11,12. New techniques have been developed to increase the ADR, such as high-definition colonoscopes 13, chromoendoscopy 14, G-EYE colonoscope 15, the Third Eye colonoscope 16, full spectrum endoscopy 17, water-immersion colonoscopy 18, retroflexion in the right colon 19, cap-assisted colonoscopy 20, endocuff-assisted colonoscopy 21, and endorings 22.

It has been suggested that colonic peristalsis may hinder the analysis of the mucosal surface, and consequently the discovery of colonic lesions.

Hyoscine is a spasmolitic agent of the gastrointestinal tract. The peripheral anticholinergic action of hyoscine results from the blockade of the intramural ganglia of hollow viscera, as well as an anti-muscarinic activity. The antiperistaltic effect of this compound on the small intestine was demonstrated by Gutzeitet et al. 23, with an average onset of action at 85 seconds after intravenous infusion and an average duration of 21 minutes. The administration of hyoscine during colonoscopy could be an alternative in controlling colonic contractility and could in this way facilitate the detection and characterization of colonic lesions. However, anti-spasmodic agents such as glucagon 24 and atropine 25 have not shown benefits in this setting. Hyoscine is inexpensive, widely available and safe. Furthermore, there have been no studies to date evaluating the use of spasmolytic agents in the differential diagnosis of colorectal lesions by digital chromoendoscopy. It was hypothesized that the analysis of the capillary pattern of colorectal lesions would be facilitated under the effect of hyoscine, which decreases the peristaltic movements of the colon.

The goal of this prospective, randomized, placebo-controlled double-blinded trial was to analyse whether the use of hyoscine enhances the detection of polyps and adenomas. As a secondary objective, this study aimed to evaluate whether this drug has any impact on the differential diagnosis of colorectal lesions during colonoscopy with magnification and digital chromoscopy.

## METHODS

The participating institutions were as follows: Santa Casa de Caridade de Bagé, RS; Evangelic Faculty of Parana, Curitiba, PR; and Santa Casa Hospital, Porto Alegre, RS. The study was approved by the Ethics Committee of Santa Casa de Caridade, Bagé, RS and was conducted according to the Declaration of Helsinki. Informed written consent 26 was obtained from all participants.

Patients who were referred to our endoscopy unit for CRC screening, surveillance or a clinical suspicion of CRC were randomly assigned to receive 20mg of hyoscine (Buscopan, Boehringer Ingelheim of Brazil Quim. e Farm. Ltda, Itapecerica da Serra, Brazil) or placebo (saline solution) as soon as the colonoscope reached the cecum. The randomization was generated by the www.researchrandomizer.org site. Sealed envelopes were opened by the nurse taking care of the patient’s sedation. The endoscopist was blinded to the randomization. The exclusion criteria were as follows: inadequate bowel preparation, incomplete colonoscopy, inflammatory bowel disease, advanced cancer, prior colorectal surgery, reported or known allergy to hyoscine, and patients referred to polypectomy or endoscopic mucosal resection due to previously diagnosed lesions.

Between March and July 2015, 517 consecutively performed colonoscopies were analysed for inclusion in the study. A total of 77 cases were excluded: 20 had previously diagnosed colon lesions and were referred for colonoscopic removal; 13 had previously undergone colon resection surgery; 11 had inadequate bowel preparation; 7 had advanced carcinomas; 7 were aged less than 30 years; 7 being allergic to hyoscine and in 2 cases the cecum was not reached. Therefore, 440 cases were randomized, with 220 cases in each arm. The patients were analysed with respect to age, gender, colonoscope withdrawal time, endoscopic findings and histopathology.

### Procedures

Bowel preparation was undergone with a clear liquids diet on the day prior to the procedure and 1000 ml of a 10% mannitol solution on the day of lower endoscopy. All colonoscopies were performed under conscious sedation with midazolamand fentanyl.

One millilitre of hyoscine (20 mg) or placebo was infused intravenously as soon as the cecum was reached during colonoscopy (Fujinon 590ZW5 with high resolution and magnification, Fujifilm Corp., Saitama, Japan) with the EPX4400 processor. We waited for at least one minute before starting withdrawal, of the coloscope because the antispasmodic effect begins within one minute and lasts for 10-15 minutes 27.

All procedures were performed by one of the authors (CEOS) who had already performed more than 12,000 colonoscopies with magnification and real or digital chromoendoscopy.

The colonoscope withdrawal time was defined as the time spent between cecum examination after the intravenous administration of the drug or placebo and the removal of the colonoscope through the anus. In all cases, this procedure lasted more than 6 minutes.

Patients were monitored continuously during the examination by means of a pulse oximeter. The alarm remained off during the colonoscopies and the monitor was seen only by the nurse and not by the endoscopist. To maintain patient safety, the nurse was instructed to communicate with the endoscopist in case of significant tachycardia, which was considered relevant when the heart beat rate was greater than 140/minute for more than 30 seconds 28.

### Lesions characteristics

The lesion size was estimated with an open biopsy forceps (7mm). The lesions morphology was determined according to the Paris classification 29, in which non-polypoid lesions are considered those less than 2.5mm in height, which was estimated with a closed biopsy forceps touching the lateral margins of the lesion, (types 0-IIa, 0-IIa+IIc, 0-IIc+IIa, 0-IIc, and 0-IIb and laterally spreading tumors - LSTs). Polypoid lesions are those greater than 2.5mm in height (types 0-Is, 0-Isp, and 0-Ip). All lesions included in the study had the endoscopic appearance of lesions known as superficial lesions, limited to the mucosa or submucosa, according to Kudo 30. The right colon was considered the cecum, and the ascending and transverse colon, while the left colon was the rectum, the sigmoid and the descending colon.

All lesions were evaluated using digital chromoendoscopy (Flexible Spectral Imaging Color Enhancement – FICE) to analyse capillary patterns for the real time differential diagnosis between neoplastic and non-neoplastic lesions.

According to the Teixeira Classification 31, lesions with I-II patterns were considered non-neoplastic, and lesions with III-V patterns were considered neoplastic. Non-neoplastic lesions were hyperplastic and inflammatory polyps, and neoplastic lesions were tubular, tubulovillous, villous, sessile serrated, and traditional serrated adenomas and early carcinomas. Those lesions greater than 1 cm, with villous histology or high-grade dysplasia were considered advanced adenomas. Advanced neoplasias were the advanced adenomas and early carcinomas.

All the lesions were removed endoscopically and were analysed by the same pathologist who was blinded to the endoscopic diagnosis.

### Statistical Analysis

The data were inserted into the Stata software version 11.2. Categorical variables were described using absolute and relative frequencies. Numerical variables were described as the mean and standard deviation (SD) when the distribution of the data was normal or as the median and interquartile interval when their distribution was not normal.

Bivariate analyses of the categorical variables comparing the hyoscine and placebo groups were performed using Fisher’s exact test. Bivariate analyses of numerical variables comparing the groups with and without hyoscine were performed using a t-test (when comparing means) or the Mann-Whitney U test (when medians were compared).

A logistic regression was used to analyse the adenoma detection rate (ADR) with the odds ratio (OR) and confidence interval (95% CI). For the sample size calculation, the analysis of the detection rate of adenomas was considered as an outcome, stratified according to the treatment group (hyoscine) and control group (placebo). The parameters considered were as follows: power of 80%, alpha-error of 5%, prevalence of 50% outcome, 10-75% exposure frequency and relative risk of at least 1.7 or an odds ratios of 2.6 between exposed *versus* unexposed, resulting in a patient number of 216.

The sensitivity, specificity, positive predictive value (PPV) and negative predictive value (NPV) of the capillary pattern analysis were reported. The significance level adopted was 5% for the bicaudal tests.

## RESULTS

From a total of 517 consecutive patients, 77 (14.9%) did not meet the inclusion criteria. Therefore, 220 patients were randomized to the hyoscine group, and the other 220 were included in the placebo group. A total of 563 lesions were diagnosed in 287 colonoscopies (65.2%), with 391 adenomas in 217 patients (49.3%). These figures are described in Table 1.

Sex, age, mean lesion size (4.9 mm +/- 4.2 *vs* 5.3 mm +/- 6.2, *p*=0.46), and mean colonoscope withdrawal time (9.8 min +/- 5.7 *vs* 9.8 min +/- 8.3, *p*=0.99) were similar between the two groups. However, the median and the interquartile interval were higher in the placebo group with respect to the colonoscope withdrawal time [7.7 min (7.1-9.8) *vs* 7.5 min (6.8-8.6), *p*=0.05].

In the hyoscine group, significantly more non-polypoid lesions were detected than in the placebo group (79.8% *vs* 69.4%); nevertheless, polypoid lesions composed 20.2% of the lesions in the hyoscine group and 30.6% in the placebo group (*p*=0.01). The proportion of adenomas and non-polypoid adenomas found in the right colon was similar between the two groups.

Analysing the lesions via digital chromoendoscopy in the placebo group, the accuracy was 94.3%, the sensitivity 94.1%, the specificity 94.7%, the PPV 98% and the NPV 85.7%, while these values were 95%, 93.7%, 97.8%, 98.9% and 88.1%, respectively, in the hyoscine group (Table 2, all these data were not significantly different).

The detection of advanced adenomas (12.7% *vs* 11.8%) and advanced neoplasias (13.6% *vs* 12.3%), the PDR (64.6% *vs* 65.9% ) and the ADR (50.5% *vs* 46.8%) were also similar between the placebo and the hyoscine groups, respectively (Table 1), as well as the mean number of polyps (1.3 *vs* 1.3), adenomas (0.92 *vs* 0.86), advanced adenomas (0.16 *vs* 0.18) and advanced neoplasias (0.16 *vs* 0.18) detected per patient (*p*=0.97).

The logistic regression analysis demonstrated that 1 additional minute of colonoscope withdrawal time and the presence of one more lesion were associated with the detection of one more adenoma in the placebo group, and the presence of a lesion in the right colon was associated with the detection of more lesions in the hyoscine group.

Polypoid lesions were a risk factor for adenoma only in the placebo group. The complete logistic regression analysis is depicted in Table 3.

No serious side effects were observed in the study groups. Three patients presented with significant tachycardia lasting less than 30 seconds in the hyoscine group.

## DISCUSSION

We hypothesized that the spasmolytic action of hyoscine would favour a greater detection of polyps and adenomas, thus increasing the ADR. Other studies have suggested that the administration of a spasmolytic drug could also facilitate the insertion of the colonoscope to the cecum 32,33 or could improve the visualization of the colonic surface due to the reduction of spasms and the flattening of the colonic haustrations 34. However, these results were not reproduced by other authors 35,36.

Employing a grading scale for colonic spasms, Lee et al. 37 observed a significant decrease in the number of spasms between colonoscope insertion and withdrawal in patients receiving hyoscine in comparison to those receiving placebo. However, this group did not show any difference in the number of polyps per patient or the ADR. Nonetheless, Lee et al. found a trend for a higher polyp detection rate in the hyoscine group when comparing a subgroup of placebo patients with severe spasms. Our study did not show an advantage to injecting hyoscine when the colonoscope reached the cecum in terms of the PDR or ADR, which is in line with two recently published metanalyses 38,39. There was also no diagnostic advantage either in the detection of advanced adenomas, or advanced neoplasias.

In a study of 601 patients, Corte et al. 40 identified a higher polyp rate per patient in a hyoscine group than in a placebo group (0.91 *vs* 0.7 *p*=0.04), but the PDR and ADR were similar for both arms (43.6 *vs* 36.6% and 27.1% *vs* 21.8%, respectively). In a study by de Brouwer et al. 41, there was no difference in the PDR, ADR or mean number of polyps detected per patient, in an Italian double-blind, randomized trial comparing hyoscine and placebo 28.

The present study also did not demonstrate any difference in the PDR, ADR, or mean number of detected polyps or adenomas per patient when comparing hyoscine with placebo.

In contrast to the findings of Rondonotti et al. 28, who encountered significantly fewer non-polypoid colorectal lesions in the hyoscine group, we found significantly more non-polypoid colorectal lesions in the hyoscine arm. Our findings refute the hypothesis that spasmolytic agents would hinder the identification of flat lesions by stretching of the colon. However, de Brouwer et al. 41 identified no difference in the morphology of the diagnosed colorectal lesions, when comparing hyoscine and placebo.

We observed a longer withdrawal time in the placebo group, which in our study, was associated with the ADR according to the logistic regression analysis. In contrast, in the hyoscine group, the main factor associated with the ADR was the detection of a lesion in the right colon. These figures are comparable to those reported by Corte et al. 40. Perhaps this longer withdrawal time in the placebo group can be explained by waiting for the colonic spasms to end during the examination.

The analysis of the capillary pattern by magnification colonoscopy with digital chromoscopy has yielded excellent results in the differential diagnosis of neoplastic and non-neoplastic colorectal lesions, with good to excellent intra- and interobserver agreement 42-45. We also hypothesized that the abolishment of colonic contractility would allow a better evaluation of these lesions by digital chromoendoscopy with magnification. However, no differences regarding the diagnostic sensitivity, specificity, PPV, NPV or accuracy were observed when comparing patients who received placebo or hyoscine. Perhaps, the use of hyoscine could improve the discrimination between neoplastic and non-neoplastic colorectal lesions, and improve the PDR and ADR among beginners, but not after the acquisition of expertise. Indeed, one of the limitations of this study is that all the examinations were performed by a single endoscopist, who is very familiar with the Japanese Classification of Colorectal Carcinoma 46, which may explain the large number of lesions morphologically classified as NPLs.

In summary, we found a higher number of non-polypoid lesions in the hyoscine group and more polypoid lesions in the placebo group, but with no difference with respect to the colonic segment (left or right). This double-blind, prospective, placebo-controlled trial did not show any evidence supporting the routine use of hyoscine during colonoscopy to improve the PDR and ADR or to augment the diagnosis of advanced adenomas or advanced neoplasias. We could also not demonstrate an impact of the drug in differentiating neoplastic from non-neoplastic lesions by means of digital chromoendoscopy with magnification.

## AUTHOR CONTRIBUTIONS

Santos CE, Pereira-Lima JC, Onófrio FQ were involved in subjects recruitment, data collection, interpretation of the results, provided assistance in statistical analyses and manuscript drafting. Moreira H, Ribas CA reviewed the manuscript, conceived and designed the study. Czeczko AE, Ramos RK, de Carvalho CA were involved in data collection, literature review and contributed to the results.

## Figures and Tables

**Table 1:** Patient and lesion characteristics.Patient and lesion characteristics.

Variable	Placebo group (%)	Hyoscine group (%)	*p* value*
	N=220 patients	N=220 patients	
Sex			0.76
Female	151 (68.6)	147 (66.8)	
Male	69 (31.4)	73 (33.2)	
Age (years)			0.50
<50	47 (21.4)	54 (24.6)	
≥50	173 (78.6)	166 (75.4)	
Lesions per patient			0.65
0	78 (35.4)	75 (34.0)	
1	68 (30.9)	71 (32.3)	
2	38 (17.3)	42 (19.1)	
3	24 (10.9)	16 (7.3)	
≥4	12 (5.5)	16 (7.3)	
Size (mm)	N= 281 lesions	N=282 lesions	1.00
<10	262 (93.2)	262 (92.9)	
≥10	19 (6.8)	20 (7.1)	
Morphology			0.01
Non-polypoid	195 (69.4)	225 (79.8)	
Polypoid	86 (30.6)	57 (20.2)	
Capillary pattern			0.15
Non-neoplastic	84 (29.9)	101 (35.8)	
Neoplastic	197 (70.1)	181 (64.2)	
Pathology			0.20
Non-neoplastic	76 (27.1)	91 (32.3)	
Neoplastic	205 (72.9)	191 (67.7)	
Adenomas			0.23
No	79 (28.1)	93 (33.0)	
Yes	202 (71.9)	189 (67.0)	
Advanced adenoma			0.62
No	247 (87.9)	243 (86.2)	
Yes	34 (12.1)	39 (13.8)	
Advanced neoplasia			0.71
No	245 (87.2)	242 (85.2)	
Yes	36 (12.8)	40 (14.8)	
Advanced histology			0.45
No	265 (94.3)	270 (95.7)	
Yes	16 (5.7)	12 (4.3)	
Location			0.18
Right Colon	152 (54.1)	136 (48.2)	
Left Colon	129 (45.9)	146 (51.8)	

**Table 2:** Diagnostic criteria of the lesions using digital chromoendoscopy.

	Placebo group *(n=281)*	Hyoscine group *(n=282)*
Sensitivity (%)	94.1 (90.0 - 95.9)	93.7 (89.3 - 95.7)
Specificity (%)	94.7 (87.1 - 98.5)	97.8 (92.3 - 99.7)
PPV (%)	98.0 (94.9 - 99.4)	98.9 (96.1 - 99.9)
NPV (%)	85.7 (76.4 - 92.4)	88.1 (80.2 - 93.7)
Accuracy (%)	94.3	95
*Kappa*	0.86 (0.79 - 0.93)	0.89 (0.83 - 0.95)

**Table 3:** Logistic regression analysis of the adenoma detection rate.

	Placebo group		Hyoscine group	
Variable	OR IC95%	*p* value	OR IC95%	*p* value
Male sex	1.4 (0.8-2.4)	0.3	1.2 (0.7-2.2)	0.5
≥50 years	2.8 (1.4-5.5)	<0.01	2.4 (1.3-4.6)	0.01
Leions (number)	24 (19-60)	<0.01	7 (4.2-12)	<0.01
Polypoid lesions	8.3 (1.9-35)	<0.01	1.7 (0.7-3.7)	0.2
Right colon	1.8 (0.9-3.7)	0.1	5.8 (2.8-12)	<0.01
Time (min)	3.6 (2.4-5.4)	<0.01	2.0 (1.5-2.5)	<0.01
